# *NCAPG2* Maintains Cancer Stemness and Promotes Erlotinib Resistance in Lung Adenocarcinoma

**DOI:** 10.3390/cancers14184395

**Published:** 2022-09-09

**Authors:** Shiyao Jiang, Jingjing Huang, Hua He, Yueying Liu, Lu Liang, Xiaoyan Sun, Yi Li, Li Cong, Bei Qing, Yiqun Jiang

**Affiliations:** 1 The Key Laboratory of Model Animal and Stem Cell Biology in Hunan Province, Hunan Normal University, Changsha 410013, China; 2 School of Medicine, Hunan Normal University, Changsha 410013, China; 3 Department of Thoracic Surgery, The Second Xiangya Hospital, Central South University, Changsha 410013, China

**Keywords:** erlotinib resistance, cancer stem cells, lung adenocarcinoma, tumor microenvironment, *NCAPG2*

## Abstract

**Simple Summary:**

This study investigated the relationship between erlotinib resistance and stemness in lung adenocarcinoma. *NCAPG2* was identified as an erlotinib resistance gene and maintained the stemness of lung adenocarcinoma.

**Abstract:**

Erlotinib is a highly specific and reversible epidermal growth factor receptor tyrosine kinase inhibitor (EGFR-TKI), but resistance inevitably develops as the disease progresses. Erlotinib resistance and cancer stem cells (CSCs) are poor factors hindering the prognosis of patients with lung adenocarcinoma (LUAD). Although studies have shown that erlotinib resistance and CSCs can jointly promote cancer development, the mechanism is currently unclear. Here, we investigated the potential biomarker and molecular mechanism of erlotinib resistance and cancer stemness in LUAD. An erlotinib resistance model based on four genes was constructed from The Cancer Genome Atlas (TCGA), the GEO database, the Cancer Cell Line Encyclopedia (CCLE), and the Genomics of Drug Sensitivity in Cancer (GDSC). Through multiple bioinformatic analyses, *NCAPG2* was identified as a key gene for erlotinib resistance and stemness in LUAD. Further in vitro experiments demonstrated that *NCAPG2* maintains stemness and contributes to erlotinib resistance in LUAD. In summary, *NCAPG2* plays a vital role in stemness and erlotinib resistance in LUAD.

## 1. Introduction

Lung cancer is one of the most lethal malignancies in the world, leading to a global public health problem [1]. Non-small cell lung cancer (NSCLC) accounts for 85% of lung cancers, which is more than half of lung cancers; lung adenocarcinoma (LUAD) is the major subtype of NSCLC, with a poor prognosis and a tendency to recur and metastasize. Although there are significant advances in small-molecule targeted therapy using epidermal growth factor receptor tyrosine kinase inhibitors (EGFR-TKIs), the emergence of acquired drug resistance has led to persistently low 5-year survival rates in LUAD [2]. Therefore, there is an urgent need to investigate the molecular mechanisms of erlotinib resistance in LUAD and to find effective therapeutic targets.

Erlotinib is a highly specific and reversible EGFR-TKI, primarily applied in metastatic NSCLC with tumor epidermal growth factor receptor (EGFR) exon 19 deletion or exon 21 (L858R) mutation 9 [3]. Erlotinib has become the first-line treatment for LUAD with EGFR mutations [4]. Using erlotinib significantly prolonged the survival of patients with LUAD [5]. However, the clinical outcome of some patients was not significantly improved due to the emergence of acquired resistance [6]. Two main reasons for the acquired resistance to erlotinib in LUAD patients were found. Firstly, the secondary mutation of EGFR enhances the binding of ATP to EGFR, reducing the effect of EGFR-TKI, which acts by competing for ATP binding [7]. Secondly, erlotinib resistance could also be caused by the abnormal activation of c-MET amplification, the abnormal expression or mutation of HER2 [8], the abnormal activity of the downstream EGFR pathway [9], and K-Ras mutation [10,11]. Recent evidence shows that cancer stemness is correlated with tumorigenesis, tumor development, and resistance to chemotherapeutic drugs [12,13]. However, the mechanism between erlotinib resistance and cancer stemness in LUAD currently requires further study.

Cancer stem cells (CSCs) undergo unlimited proliferation and multi-directional differentiation, significantly challenging cancer treatment [12]. Recent studies have shown CSCs to play a role in tumor drug resistance. The exposure of glioma cell lines to therapeutic doses of temozolomide (TMZ) consistently increases the glioma stem cell pool over time, both in vitro and in vivo [14]. Besides, a drug-resistant subline of LUAD with stem-like properties is resistant to conventional chemotherapeutics [14]. Therefore, it is of great interest to explore the relationship between erlotinib resistance and stemness in LUAD to help identify new therapeutic targets for erlotinib-resistant patients.

Previous studies have shown that the tumor microenvironment, the survival environment of the tumor, is composed of various cells [15]. Immune cells in the tumor microenvironment play an essential role in regulating the development of drug resistance [16,17] and supporting cancer stem cells [18]. Tumor-associated fibroblasts support the stemness of CSCs through paracrine mechanisms. The factors secreted by stromal cells can induce microenvironment-mediated drug resistance [15]. LUAD cell lines were resistant to EGFR-TKIs when co-cultured with cancer-associated fibroblasts expressing podoplanin [19]. These findings suggest that the tumor microenvironment is essential for the progression of LUAD.

Non-SMC condensin II complex subunit G2 (*NCAPG2*), a regulatory subunit of the condensin II complex, is involved in chromosome assembly and segregation during mitosis [20]. Studies have shown that *NCAPG2* is vital in regulating proper chromosome segregation during mitosis through a functional interaction with polo-like kinase 1 (PLK1) [21]. *NCAPG2* promotes tumor proliferation by regulating the G2/M phase and is associated with poor prognosis in LUAD [22]. However, the role of *NCAPG2* in erlotinib resistance and stemness of LUAD requires further study.

This study used The Cancer Genome Atlas (TCGA), the GEO database, the Cancer Cell Line Encyclopedia (CCLE), and the Genomics of Drug Sensitivity in Cancer (GDSC) databases to identify candidate genes associated with erlotinib resistance and cancer stemness in LUAD. An erlotinib resistance model was constructed using candidate genes. Furthermore, the correlation between erlotinib resistance genes and stemness was analyzed through various bioinformatics methods. We also examined the correlation between the erlotinib resistance genes and the tumor microenvironment. Finally, in vitro experiments demonstrated that *NCAPG2* maintains cancer stemness and promotes erlotinib resistance in LUAD cells. This study provides potential therapeutic strategies for patients with erlotinib-resistant LUAD.

## 2. Materials and Methods

### 2.1. Data Collection

The LUAD erlotinib resistance dataset GSE31625 (https://www.ncbi.nlm.nih.gov/geo/query/acc.cgi?acc=GSE31625 (accessed on 23 March 2022)) and the stemness-related dataset GSE35603 (https://www.ncbi.nlm.nih.gov/geo/query/acc.cgi?acc=GSE35603 (accessed on 23 March 2022)) from the GEO database were used. Cell line details, including IC50 and the gene expression profiles of the cells, were downloaded from the Cancer Cell Line Encyclopedia (CCLE, https://portals.broadinstitute.org/ccle/data (accessed on 23 March 2022)) and the Genomics of Drug Sensitivity in Cancer (GDSC, https://www.cancerrxgene.org/ (accessed on 23 March 2022)). We collected the gene expression profiles and clinical information of 496 LUAD patients from The Cancer Genome Atlas (TCGA, https://gdc.cancer.gov/ (accessed on 23 March 2022)); GSE30219 (https://www.ncbi.nlm.nih.gov/geo/query/acc.cgi?acc=GSE30219 (accessed on 23 March 2022)) was utilized to collect the gene expression profiles and clinical information from 77 LUAD patients; similarly, the gene expression profiles of 58 patients with LUAD were collected from GSE10072 (https://www.ncbi.nlm.nih.gov/geo/query/acc.cgi?acc=GSE10072 (accessed on 23 March 2022)), and the gene expression profiles of 442 patients with LUAD were obtained from GSE72094 (https://www.ncbi.nlm.nih.gov/geo/query/acc.cgi?acc=GSE72094 (accessed on 23 March 2022)). GSE31625, GSE35603, GSE30219, GSE10072, and GSE72094 were from the GEO cohort.

### 2.2. Variance Analysis

Differentially expressed genes (DEGs) between erlotinib-resistant and sensitive groups from GSE31625 and CD133^+^ versus CD133^−^ from GSE35603 were analyzed using the R package ‘limma’ with the screening criteria |FC| ≥ 1.5 and *p* < 0.05. The R package ‘pheatmap’ was used to generate volcano maps to represent the results of the variance analysis. The R package ‘VennDiagram’ and univariate Cox regression analysis were used to identify the candidate genes.

### 2.3. Erlotinib-Resistant-Related Model Construction

The CCLE and GDSC data were processed using LASSO regression and the R package ‘glmnet’ to obtain erlotinib resistance-associated mRNAs by the penalty of the maximum likelihood fitness. Based on the results of LASSO, the glmnet R package was used to select variables and build a predictable resistance model. ROC curves were analyzed with the R package ‘timeroc’ and visualized with ‘ggplot2’.

### 2.4. GO, KEGG, and GSVA Analysis

Gene Ontology (GO), the Kyoto Encyclopedia of Genes and Genomes (KEGG), and Gene Set Variation Analysis (GSVA) [23] were performed to obtain the biological significance related to erlotinib resistance. The R package ‘clusterProfiler’ was used for the GO and KEGG analysis, and the R package ‘GSVA’ for GSVA.

### 2.5. The Stemness Index Based on mRNA Expression

The mRNA expression-based stemness index (mRNAsi) of LUAD patients in TCGA, GSE10072, and GSE72094 was obtained from R language-based machine learning code in the article of M. Malta [24]. The correlation between gene expression and mRNAsi was calculated by ‘corrplot’ and visualized by the ‘ggplot2’ package.

### 2.6. Constructing a Prognostic Model

The genes related to erlotinib resistance and stemness were screened and analyzed by multivariate analysis to determine the prognosis model. The patients’ risk score was identified according to the gene expression levels in TCGA and GSE35603 and the regression coefficient of Cox. Kaplan–Meier (KM) survival curves were plotted using the R packages ‘survival’ and ‘Survivor’.

### 2.7. Immune Analysis

The fraction of 20 immune cells was calculated using CIBERSORTx [25]. The immune purity score was calculated through ESTIMATE. The correlation between the genes and the immune cells was calculated using the algorithm single-sample Gene Set Enrichment Analysis (ssGSEA) built into the software package ‘GSVA’.

### 2.8. Single-Cell RNA Sequencing (scRNA-Seq) Analysis

A routine data integration process was performed on samples from GSE134839 and GSE134841 using the R package ‘Seurat’. Quality control and filtering of the dataset were performed on the integrated Seurat objects. Single cells with less than 200 or more than 4000 unique molecular identifiers (UMIs) or with more than 10% of the mitochondria-derived UMI counts were considered low-quality cells and were removed. The ‘LogNormalize’ method in the ‘NormalizeData’ function normalizes the raw counts and uses the ‘FindVariableFeatures’ method to identify highly variable genes. ‘ScaleData’, a linear transformation function, was then used to ensure that the expression of all the genes had the same weight in the downstream analysis and that highly expressed genes did not dominate. The data were then subjected to principal component analysis (PCA) to reduce the dimensionality of the data. The ‘FindNeighbors’ and ‘FindClusters’ functions performed cell clustering and were visualized using uniform manifold approximation and projection (UMAP).

### 2.9. Cell Culture and Plasmids

Human lung adenocarcinoma cell lines PC9 and HCC827 were purchased from the American-Type Culture Collection (ATCC). The cells were cultured in RPMI 1640 medium (Gibco, China), containing 10% fetal bovine serum (FBS, Biological Industries, Israel) at 37 °C and 5% CO_2_. Erlotinib-resistant cell lines (PC9-ER and HCC827-ER) were generated by gradually increasing the erlotinib concentrations in a continuous culture of PC9 and HCC827 for approximately one and a half years. These cells were considered resistant until the parental cells could grow at a concentration of 2μM [26]. Lentiviral short hairpin RNA (shRNA) vectors targeting human *NCAPG2* and a nontargeted control vector were constructed by Genechem (https://www.genechem.com.cn (accessed on 1 June 2022); Shanghai, China). Plasmid transfection was performed using packaging plasmid and ExFectTransfection Reagent (Vazyme, Nanjing, China), following the manufacturer’s instructions. The supernatant-containing virus was collected to infect HCC827-ER and PC9-ER, and the colonies with a stable knockdown of *NCAPG2* were screened on puromycin.

### 2.10. Cell Viability Assay

The cells were seeded into 96-well plates at a density of 2000 cells/well. Twenty-four hours after seeding, the cells were treated with increasing concentrations of erlotinib for 72 h. Ten microliters per well of CCK-8 reagent (Vazyme, China) was added to the cells and incubated for 2 h at 37 °C. Absorbance at 450 nm was measured using a Synergy 2 microplate reader (BioTek, USA). Finally, the IC50 values of erlotinib in PC9, HCC827, PC9-ER, HCC827-ER, PC9-ER sh*NCAPG2*, and HCC827-ER sh*NCAPG2* were visualized using GraphPad Prism (v 9.1.0) (San Diego, CA, America).

### 2.11. Western Blotting

The cells were lysed with IP lysis buffer (Thermo, Waltham, MA, USA), and the protein concentrations were determined using a BCA kit (Vazyme, China). The proteins were then denatured with sodium dodecyl sulfate (SDS). Electrophoresis was performed on a 10% polyacrylamide gel in an SDS loading buffer. The gel was run at 90 V for 30 min and then switched to a 130 V run until the end of the electrophoresis. Subsequently, the proteins were transferred to polyvinylidene fluoride (PVDF) membranes (Biosharp, Hefei, China). The PVDF membranes were sealed with 10% skim milk for 2 h and then incubated overnight at 4 °C with primary antibodies, including *β-actin* (#AF7018, Affinity, Tokyo, Japan), *NCAPG2* (#ab70350, Abcam, Cambridge, UK), *EPCAM* (Abcam, ab223582), *ABCC1* (Proteintech, 67228-1-Ig, Rosemont, IL, USA), and *MYC* (Proteintech, 16286-1-AP). The membranes were incubated with secondary antibodies (#S0001, Affinity) for 1 h and finally exposed with a luminescence kit (Vazyme, China). The final visualization was carried out using a chemiluminescence system (Tanon-5200, Shanghai, China).

### 2.12. Quantitative Real-Time PCR (RT-PCR)

One milliliter of Trizol reagent (R401-01, Vazyme, Nanjing, China) was added to the target cells for lysis to obtain RNA. The HiScript^®^ II Q RT SuperMix for RT-PCR (+gDNA wiper, R223-01, Vazyme, Nanjing, China) was used to synthesize the first-strand cDNA. Quantitative PCR was performed using a real-time PCR System (CFX Connect, Bio-Rad, Hercules, CA, USA) with MonAmp™ ChemoHS qPCR Mix (MQ00401S, Monad, Shanghai, China). The primers for this experiment were derived from Sangon (Shanghai, China). Table S1 lists the primer sequences.

### 2.13. Tumorsphere Formation

Each well of an ultra-low adsorption twelve-well plate had 2000 cells added to it and was incubated at 37 °C and 5% CO_2_ for 7–14 days. The number of tumorspheres larger than 50 μm in diameter was recorded under the microscope for statistical analysis.

### 2.14. Colony Formation

About 200 control cells or the stable knockdown of *NCAPG2* for PC9-ER and HCC827-ER cells were inoculated into six-well plates and incubated for two weeks in an incubator with 5% carbon dioxide at 37 °C. The cells were then fixed with 10% methanol and stained with 0.1% crystal violet.

### 2.15. Side Population (SP) Detection by Flow Cytometry

A total of 1 × 10^6^ cells were added to three tubes. Hoechst 33,342 was added to one of the tubes to adjust the final concentration to 5 μg/mL. Hoechst 33,342 (final concentration of 5 μg/mL) and Verapamil solution (final concentration of 50 μM) were added to the other two tubes. The tubes were then incubated in a 37 °C water bath for 90 min. Subsequently, the reaction was terminated by adding pre-cooled PBS into the tube. Five hundred microliters of PBS containing 2% FBS was added to the tube after centrifugation at 1500 rpm for 10 min at 4 °C, and the supernatant was discarded. Finally, Propidium Iodide (PI) was added and mixed (final concentration to 1 μg/mL) for flow cytometry. Hoechst 33342, Verapamil, and PI were purchased from Sigma-Aldrich Company (St. Louis, MO, USA).

### 2.16. Statistical Analysis

The data were expressed as mean ± standard deviation. The Student *t*-test (unpaired) was used to analyze the differences between groups. The log-rank test was applied to compare the survival of the groups. Data analysis was performed in R (v 4.1.2, R Foundation for Statistical Computing, Vienna, Austria) with a *p*-value < 0.05 considered statistically significant.

## 3. Results

### 3.1. Identification of 25 Genes Associated with Erlotinib Resistance and Cancer Stemness in Lung Adenocarcinoma

**Figure 1:**
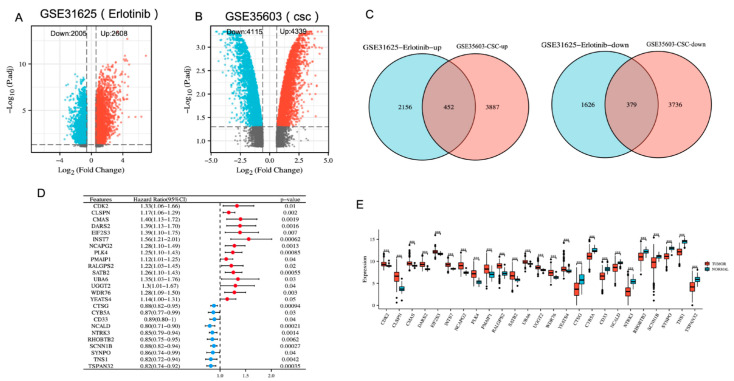
Identifying genes associated with erlotinib-resistance and stemness in LUAD. (**A**) DEGs associated with erlotinib-resistance (erlotinib-resistant cells versus sensitive cells) in LUAD from GSE31625. (**B**) DEGs associated with stemness (CD133^+^ cells versus CD133^−^ cells) in LUAD from GSE35603. (**C**) Venn diagram identifying genes associated with erlotinib resistance and stemness in LUAD. (**D**) Forest plot showing univariate regression analysis of intersecting genes. (**E**) Boxplots showing the expression of candidate genes. *** *p* < 0.001.

The data collection and analysis methods for this study are summarized in the workflow diagram shown in Figure S1. We collected 2608 upregulated and 2005 downregulated erlotinib resistance-associated DEGs (erlotinib-resistant cells versus sensitive cells) in LUAD from the dataset GSE31625 (Figure 1A). Furthermore, 4339 upregulated and 4115 downregulated cancer stem cell-associated DEGs (CD133^+^ cells versus CD133^−^ cells) in LUAD were obtained from the dataset GSE35603 (Figure 1B). Combining the DEGs from these two datasets, we obtained 452 upregulated and 379 downregulated overlapping genes associated with erlotinib resistance and cancer stemness (Figure 1C). Subsequently, these genes were analyzed using the TCGA-LUAD clinical information and expression profiles. Univariate regression analysis identified 25 genes whose expression in LUAD and normal tissue was consistent with survival risk (Figure 1D,E).

### 3.2. Construction and Validation of the Erlotinib-Resistance-Related Model

**Figure 2:**
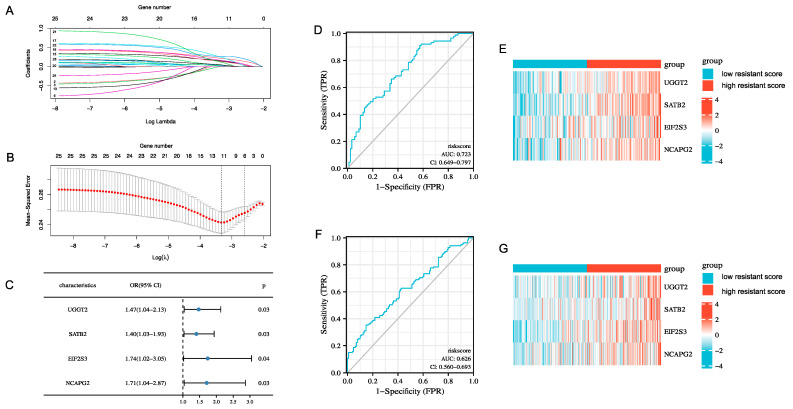
Constructing an erlotinib-resistant-related model by candidate genes. (**A**,**B**) Establishment of the erlotinib-resistant-related model. (**C**) Coefficient of logistics regression equation model of erlotinib-resistant-related genes; OR, odds ratio. (**D**) Prediction of patients’ sensitivity to erlotinib in this model using diagnostic ROC curves. (**E**) Heatmap showing gene expression in high- and low-resistance-score groups. (**F**,**G**) ROC curve and heatmap for validation group.

To explore the relationship between the 25 candidate DEGs and erlotinib resistance, we selected 497 cell lines with complete data of the mRNA expression from the CCLE database. The IC50 values for erlotinib provided in the CCLE were ranked from lowest to highest and categorized into six groups. The top 1/6 (89) with low IC50 values were defined as sensitive cell lines, and the last 1/6 (89) with high IC50 values were defined as drug-resistant cell lines. LASSO penalty analysis indicated that the minimum deviation occurred when the number of genes in the model was 11 (Figure 2A,B). Subsequently, we used logistic regression fitted to binary discrete dependent variables (glmnet R package applied to “family = binomial”) to determine whether DEG was associated with high or low IC50. From the results, it was found that 4 (*UGGT2*, *SATB2*, *EIF2S3*, and *NCAPG2*) in 11 variables had passed the significance test (*p*  <  0.05) and became relatively important variables for constructing the model (Figure 2C). The erlotinib-resistant score was calculated using the following formula: **Resistant Score** = 0.3870 × ***UGGT2***+ 0.3401 × ***SATB2***+ 0.5548 × ***EIF2S3***+ 0.5358 × ***NCAPG2***. The predictive efficiency of the erlotinib-resistant-related models was determined by constructing ROC curves (Figure 2D). With an area under the curve (AUC) of 0.723, the model we constructed, based on the four characteristic genes related to erlotinib resistance, had good accuracy for the prediction of the resistance of erlotinib. The cell lines from the CCLE database were divided into groups of high and low resistance scores based on the median resistance scores. A heat map showed that the expression of these four genes was higher in the high-resistance-score group than in the low-resistance-score group (Figure 2E). The GDSC database was used as the validation group. The receiver operating characteristic (ROC) curve had an AUC of 0.626, demonstrating a high accuracy in the erlotinib-resistant-related model of the validation group (Figure 2F). The grouping of the validation cohort was similar to that of the training cohort. The gene expression visualized using heat maps of the validation group was consistent with that of the training group (Figure 2G). The above results indicated that the model could accurately predict erlotinib resistance.

### 3.3. Functional Enrichment Analysis and Genomic Variation Analysis of the Erlotinib Resistance Model

**Figure 3:**
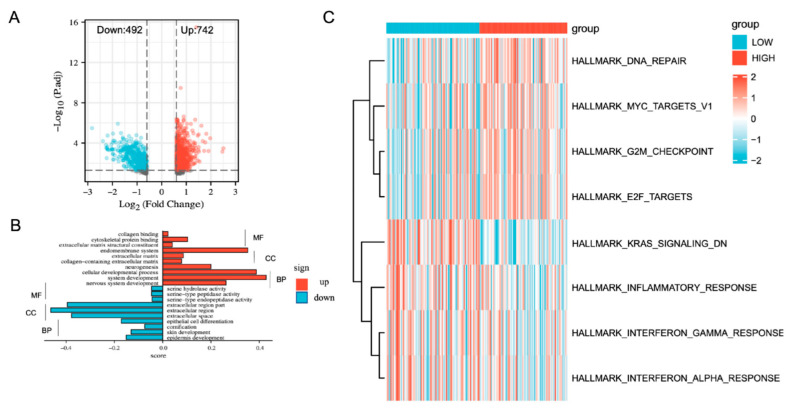
Identification of biological pathways associated with the risk of erlotinib resistance. (**A**) DEGs between the resistance score groups. (**B**) GO analysis of DEGs between the resistance score groups categorized into three functional groups: BP, CC, and MF. (**C**) GSVA of the resistance score groups. BP: biological process; CC: cellular component; MF: molecular function. Red represents upregulated genes, blue represents downregulated genes.

GO and GSVA were performed to examine the molecular mechanisms of the genes related to erlotinib resistance. The relevant samples from CCLE were divided into high- and low-resistance-score groups using the median resistant scores as the cut-off point. We performed a differential analysis between the high-resistance-score group and the low-resistance-score group (high vs. low) to obtain 742 upregulated and 492 downregulated genes in the high-resistance-score group (Figure 3A, |FC| > 1.5, *p* < 0.05). The GO analysis suggested that the upregulated genes in the high-resistance-score group were mainly enriched in biological processes related to cellular life activities, such as cell differentiation, cellular developmental processes, and system development. On the other hand, the downregulated genes in the high-resistance-score group were mainly enriched in the extracellular region part, the extracellular region, and the extracellular space (Figure 3B). Differentially expressed pathways were identified using GSVA through hallmark gene sets. The results indicated that the high-resistance-score group was related to ‘DNA repair’ and ‘MYC targets v1′, while the low-resistance-score group was associated with ‘inflammatory response’ (Figure 3C). In conclusion, we found that DEGs activate cellular life activities, ‘DNA repair’, and ‘MYC targeting v1’ pathways, which are associated with stemness and resistance. These results suggest that the erlotinib resistance model can reflect the characteristics of erlotinib resistance and tumor stemness.

### 3.4. NCAPG2 and EIF2S3 Are Positively Correlated with Cancer Stemness and Are Malignant Factors in Lung Adenocarcinoma

**Figure 4:**
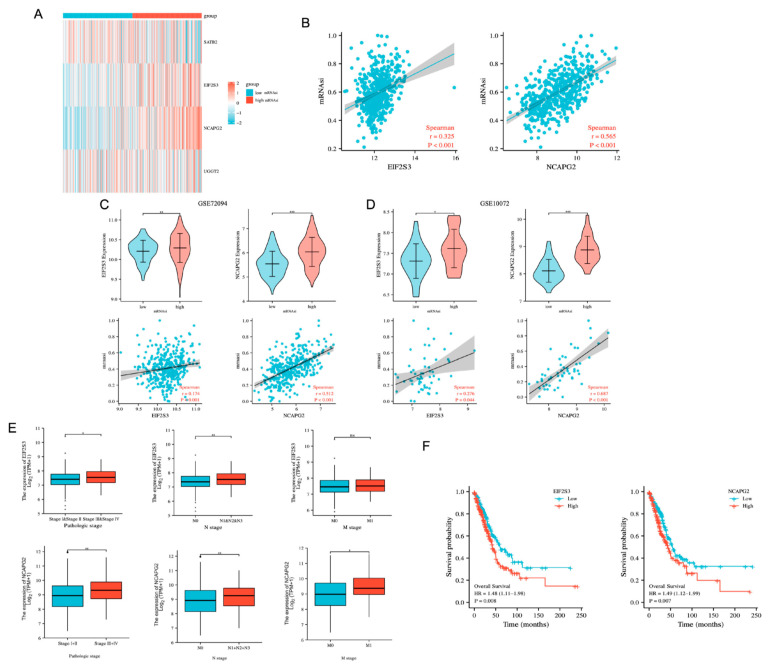
Relationship between the expression of *NCAPG2* and *EIF2S3* and stemness. (**A**) Heatmap showing gene expression in high and low mRNAsi groups. (**B**) Scatter plot showing the correlation between *NCAPG2* and *EIF2S3* and mRNAsi scores. (**C**,**D**) Validation of the association of *NCAPG2* and *EIF2S3* with stemness in LUAD from GSE10072 and GSE72094. (**E**) Relationship between *NCAPG2, EIF2S3* expression, and clinical features of LUAD. (**F**) KM survival curve showing the OS of LUAD patients with high and low expression of *NCAPG2* or *EIF2S3*. KM: Kaplan–Meier; OS: overall survival. *** *p* < 0.001, ** *p <* 0.01, * *p* < 0.05, ns: no significance.

Our GSVA results suggested that the high-erlotinib-resistance-score group was associated with the ‘DNA repair’ and ‘MYC targets v1′ pathways related to tumor stemness. Therefore, we explored the relationship between four erlotinib resistance genes and the stemness of LUAD. We selected TCGA-LUAD, GSE10072, and GSE72094 for the mRNAsi scoring. We divided TCGA-LUAD into high and low groups based on the median mRNAsi score. Among our four resistance genes, *EIF2S3* and *NCAPG2* were highly expressed in the group with high mRNAsi scores (Figure 4A). Correlation analysis revealed a positive correlation between *NCAPG2*, *EIF2S3* expression, and mRNAsi in TCGA-LUAD (Figure 4B). These outcomes were also validated in GSE10072 and GSE72094 (Figure 4C,D).

As tumor stemness is associated with tumor recurrence and metastasis, we used univariate regression to analyze the relationship between these two genes, the tumor stage, lymph node metastasis, distant metastasis, and prognosis in LUAD patients. Univariate regression analysis showed that *EIF2S3*, *NCAPG2*, the pathological stage, and lymph node metastasis were associated with the prognosis of LUAD patients. Further multiple regression analysis showed that *EIF2S3*, *NCAPG2*, lymph node metastasis, and the pathological stage (II, III, IV) interacted and were risk factors for the prognosis of LUAD (*p* < 0.05) (Table 1), suggesting that *EIF2S3*, *NCAPG2* might potentially be associated with the pathological stage and lymph node metastasis. Therefore, we used the clinical data and expression profile of TCGA-LUAD to analyze the association between the expression of *NCAPG2* and *EIF2S3*, the clinical stage, and lymph node metastasis. The results showed that *NCAPG2* expression increased with the tumor stage, lymph node invasion, and distant metastasis (Figure 4E). *EIF2S3* also increased with the tumor stage and lymph node invasion, but there was no significant change in distal metastasis. In addition, TCGA-LUAD samples were used for the KM analysis of *EIF2S3* and *NCAPG2*. The KM curve results showed that the high-expression groups of *EIF2S3* and *NCAPG2* were significantly associated with the poor prognosis of LUAD patients compared with low-expression groups (Figure 4F). These results suggested that *NCAPG2* and *EIF2S3*, as risk factors for the prognosis of LUAD patients, were positively correlated with the stemness of LUAD and promoted the development and metastasis of LUAD.

### 3.5. NCAPG2 and EIF2S3 Could Predict the Prognosis of Patients with Lung Adenocarcinoma

**Figure 5:**
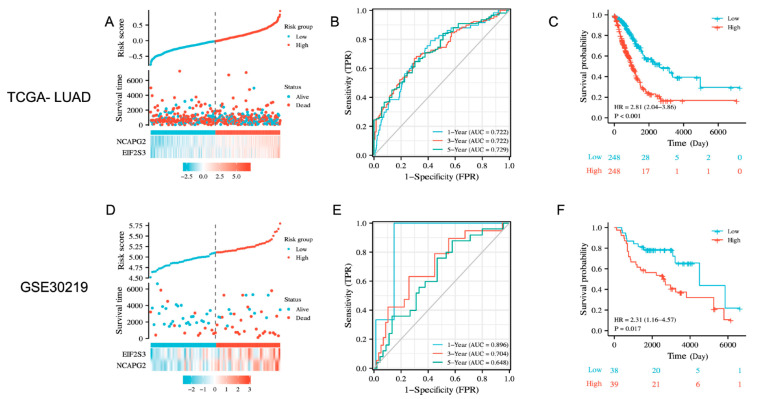
Construction and validation of a prognostic signature for *NCAPG2* and *EIF2S3* in the TCGA and GEO cohort. (**A**) Distribution of risk scores, survival status, and candidate gene expression for each patient in the high- and low-risk groups of the TCGA cohort. (**B**) AUC of time-dependent ROC curves to assess the predictive efficacy of the prognostic signature for OS in LUAD patients. (**C**) KM survival curve shows OS of LUAD patients in the high- and low-risk groups. (**D**–**F**) Validation of a prognostic signature for *NCAPG2* and *EIF2S3* in GSE30219.

A prognostic index was created based on the gene expression and the corresponding regression coefficients. The formula used is as follows: **Risk score = *NCAPG2* × 0.185082528 + *EIF2S3* × 0.436445872**. The LUAD patients from TCGA were divided into high-risk and low-risk groups based on the median risk scores. Heat maps of the risk factors showed a higher gene expression of *NCAPG2* and *EIF2S3* in the high-risk group than in the low-risk group (Figure 5A). We then evaluated the risk of the prediction models using the time-dependent ROC curves. The results showed that the AUC of ROC reached 0.722, 0.722, and 0.729 at 1, 2, and 3 years, respectively, suggesting that the prognostic model had an excellent accuracy in predicting the long-term prognosis of LUAD patients (Figure 5B). Exploring the relationship between risk score and prognosis by KM survival analysis and log-rank tests showed a worse prognosis in the patients of the high-risk group than those in the low-risk group (Figure 5C). The predictive performance and prognosis of the model were validated using GSE30219, and the results were consistent with the training cohort (Figure 5D–F). We performed a differential analysis between the high-risk score group and the low-risk score group (high vs. low) to obtain 730 upregulated and 268 downregulated genes in the high-risk-score group (Figure S2A, |FC| > 1.5, *p* < 0.05). The upregulated and downregulated DEGs in the high-risk-score group were then used for GO and KEGG analysis separately. GO analysis indicated that the upregulated genes in the high-risk group were mainly enriched in biological processes related to cellular life activities, such as nuclear division, cell division, and the mitotic cell cycle. On the other hand, the downregulated genes in the high-risk group were mainly enriched in the extracellular space and cell surface, (Figure S2B). Additionally, KEGG analysis of the DEGs between the high- and low-risk groups of the prognostic model showed that the downregulated genes in the high-risk group were enriched in the immune-related pathways, such as the cell adhesion molecules (CAMs) and the intestinal immune network for IgA production (Figure S2C), which prompted us to explore the relationship between the prognostic model and the immune system.

### 3.6. Immune Infiltration Analysis Based on the Prognostic Model

**Figure 6:**
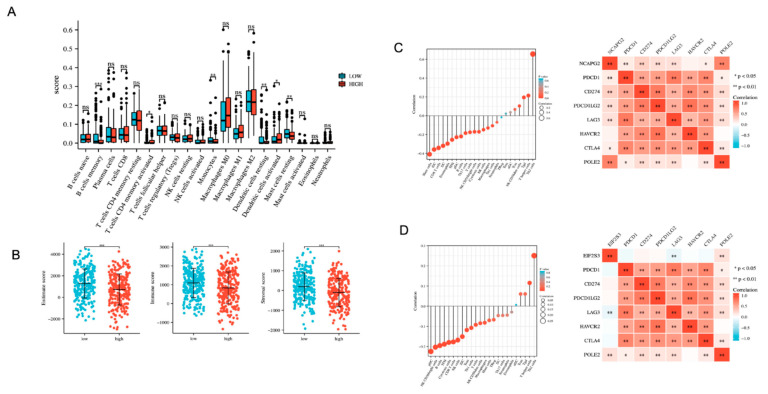
Immunoassay. (**A**) The relationship between high- and low-risk groups in the prognostic model and CIBERSORTx immune cell infiltration abundance. (**B**) The relationship between high- and low-risk groups and the immune score. (**C**) Correlation of *NCAPG2* with immune cells and immune checkpoints. (**D**) Correlation of *EIF2S3* with immune cells and immune checkpoints. *** *p* < 0.001, ** *p <* 0.01, * *p* < 0.05, ns: no significance.

To further understand the relationship between the prognosis model and the immune system, we explored how the prognosis model based on *NCAPG2* and *EIF2S3* affected LUAD by affecting the immune microenvironment. We performed CIBERSORTx on samples from TCGA-LUAD patients in the high- and low-risk groups in the prognostic model and obtained scores for 20 types of tumor-infiltrating immune cells (Figure 6A). The infiltration of immune cells such as mast cells, dendritic cells, and CD4^+^ memory T cells significantly differed between the high-risk and low-risk groups. In the low-risk group, the mast cells and dendritic cells were increased, while the CD4^+^ memory T cells were increased in the high-risk group. The association between the risk signature and the immune microenvironment of LUAD was assessed based on the ESTIMATE algorithm. The results revealed that the high-risk group in the TCGA-LUAD cohort had lower estimated immune and stromal scores than the low-risk group (Figure 6B). These results suggested that the high-risk group in the TCGA-LUAD cohort might inhibit the immune responses. We analyzed the relationship between the expression levels of *NCAPG2, EIF2S3*, immune cell infiltration, and immune checkpoints in LUAD patients in the TCGA database by ssGSEA. The results showed that the expression of *NCAPG2* was positively correlated with the infiltration of Th2 cells, T helper cells, and Tgd but negatively correlated with the mast cells, iDC, and CD8 T cells (Figure 6C, left); the expression of *NCAPG2* was positively correlated with most of the immune checkpoint pathway genes (inhibitory) (Figure 6C, right). Similarly, the expression of *EIF2S3* was positively correlated with the infiltration of Th2 cells, T helper cells, and Tgd but negatively correlated with the mast cells, iDC, and CD8 T cells (Figure 6D, left); the expression of *EIF2S3* was positively correlated with most of the immune checkpoint pathway genes (inhibitory) (Figure 6D, right). Immune checkpoint blockade therapy based on programmed death receptors and their ligands increases the host immune system’s aggressiveness against tumor cells by inhibiting the binding of the programmed death receptors and their ligands [27]. Based on the positive association of *NCAPG2* and *EIF2S3* with most of the immune checkpoint pathway genes (inhibitory), we hypothesized that high-risk populations may benefit from immune checkpoint inhibitors (ICI).

### 3.7. NCAPG2 and EIF2S3 Contributed to Erlotinib Resistance in Lung Adenocarcinoma

**Figure 7:**
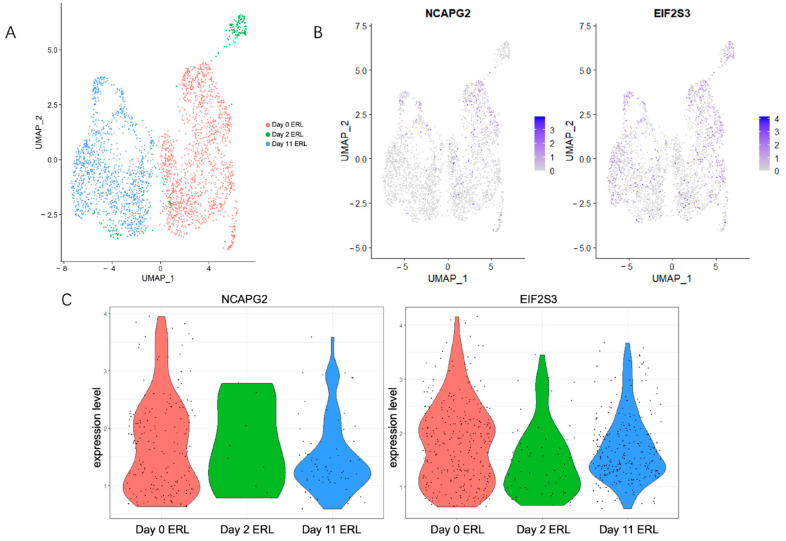
Single-cell RNA sequencing (scRNA-seq) analysis using GSE134841. (**A**) Visualization of scRNA-seq in three datasets using UMAP. (**B**,**C**) UMAP plots and violin plots showing the expression of *NCAPG2* and *EIF2S3* in different cell populations.

To detect the transcriptomic changes in *NCAPG2* and *EIF2S3* during erlotinib exposure at the single-cell level, we analyzed scRNA-seq data from the GSE134839 and GSE134841 datasets for no-dose, two-day-dose, and eleven-day-dose groups. The scRNA-seq data were visualized using UMAP, following the noise reduction procedure (see Materials and methods for details, Figure 7A and Figure S3A ). The *NCAPG2* and *EIF2S3* gene expressions were also mapped to the UMAP results to observe their abundance in each cell population (Figure 7B and Figure S3B). The violin plot visualizes the different expressions of *NCAPG2* and *EIF2S3* within these three cell populations (Figure 7C and Figure S3C). The results showed downregulation in *NCAPG2* and *EIF2S3* expression during short-term erlotinib treatment. In comparison, the expression appeared to be upregulated during the long-term erlotinib treatment relative to the short-term treatment. This phenomenon might result from cells entering the drug-tolerant persisters (DTP) state at the beginning of the drug treatment in response to intense drug exposure. The cells enter a quiescent state to escape the damage caused by the drug [28]. The knockdown of *NCAPG2* has been shown to induce cell cycle arrest at the G2 phase to inhibit LUAD cell proliferation [22]. We speculate that short-term drug treatment altered the cell cycle signaling by leaving *NCAPG2* in a low-expression state, enabling the cells to enter the DTP state. Additionally, one of the characteristics of DTP is the presence of low levels of mRNA translation [29]. *EIF2S3* is a subunit of eukaryotic initiation factor 2 (eIF2) and is involved in the initiation of protein synthesis [30]. We suggest that cells could adapt to DTP by reducing the expression of *EIF2S3* at the beginning of drug treatment, possibly reducing the ability of the cells to synthesize specific proteins. As the erlotinib treatment was extended, a small proportion of DTPs started to proliferate again, resulting in the cells entering the drug-tolerant expanded persister (DTEP) state [28] with the increased expression of *NCAPG2* and *EIF2S3*.

### 3.8. NCAPG2 Promoted Stemness and Erlotinib Resistance in Lung Adenocarcinoma Cells

Our analysis determined an association of *NCAPG2* with LUAD stemness and erlotinib resistance. Based on this, we further explored the mechanism of *NCAPG2* concerning drug resistance and stemness. The TCGA-LUAD samples were divided into high and low groups according to the median expression level of *NCAPG2*. Then, GSVA analysis found that gene sets such as the mTORC1 signaling pathway, DNA repair, and MYC targets were mainly enriched in the *NCAPG2* high-expression group. This suggested that *NCAPG2* might play a pro-tumor role through the mTORC1 signaling pathway, DNA repair, and MYC targets (Figure S4A).

**Figure 8:**
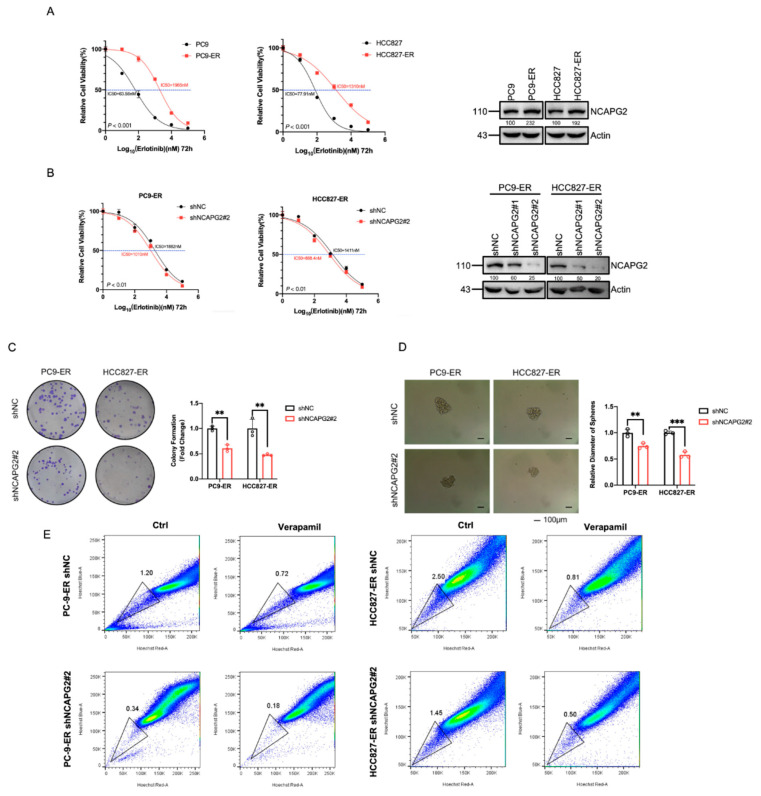
Knockdown of *NCAPG2* significantly increases sensitivity to erlotinib while reducing stemness in LUAD. (**A**) The cell viability assay revealed that resistant cell lines (PC9-ER and HCC827-ER) were constructed successfully. Western blot displays the expression of NCAPG2 in PC9-ER and HCC827-ER. (**B**) *NCAPG2* stable knockdown by PC9-ER and HCC827-ER cells was verified by Western blot and the cell viability assay. (**C**) Colony formation showed that knockdown of *NCAPG2* reduced colony formation in PC9-ER and HCC827-ER. (**D**) Tumor sphere-forming assay revealed that silencing *NCAPG2* significantly reduces the diameter of the spheres in PC9-ER and HCC827-ER. (**E**) SP detection significantly reduces the side population cells in PC9-ER and HCC827-ER upon silencing *NCAPG2*. All experiments (except flow cytometry) were repeated three times. *** *p* < 0.001, ** *p <* 0.01. All the whole Western blot figures can be found in the Supplementary Materials.

To verify the effect of *NCAPG2* on erlotinib resistance in LUAD, we constructed PC9 and HCC827 erlotinib-resistant cells and assayed the IC50 values of erlotinib individually. The cell viability assay suggested that the IC50 value of PC9-ER was 1965 nM, 30.9 times higher than the 63.56 nM of PC9, and the IC50 of HCC827-ER was 1310 nM, 16.8 more than the 77.91 nM of HCC827. This demonstrated that the erlotinib-resistant cells were successfully generated. Western blotting showed that NCAPG2 was highly expressed in ER cells, suggesting that NCAPG2 may promote resistance to erlotinib (Figure 8A). Then, we knocked down *NCAPG2* in ER cells with two independent shRNAs (sh*NCAPG2*#1 and sh*NCAPG2*#2) and assessed the knockdown efficiency using Western blotting. We infected the cells with the lentivirus containing the shRNA vector and screened for antibiotics to obtain a stable cell line, and then, the stably infected sh*NCAPG2*#2 cell line with a better knockdown effect was selected for subsequent experiments. The cell viability assay suggested that the IC50 value of PC9-ER was 1882 nM, 1.86 higher than the 1010 nM of PC9-ER sh*NCAPG2*#2, and the IC50 of HCC827-ER was 1411 nM, 1.62 more than the 868.4nM of HCC827-ER sh*NCAPG2*#2. The results demonstrated that the knockdown of *NCAPG2* enhanced sensitization of LUAD resistance cells to erlotinib treatment (Figure 8B). In addition, we performed clone-formation experiments on knockdown *NCAPG2* in PC9-ER and HCC827-ER cells and control cells. The results showed that the clone volume of PC9-ER and HCC827-ER was smaller than that of the control group after the knockdown of *NCAPG2*, suggesting that the knockdown of *NCAPG2* could inhibit the proliferation of erlotinib-resistant cells (Figure 8C). These results confirmed that *NCAPG2* promoted erlotinib resistance and tumor cell proliferation in LUAD.

To determine the effect of *NCAPG2* on the stemness of LUAD, we performed a tumor sphere-formation assay and SP detection by flow cytometry. The tumor sphere-formation assay indicated that the diameter of the spheres from the erlotinib resistance cells with *NCAPG2* knockdown was significantly smaller than that of the controls (Figure 8D). The SP cells in PC9-ER, HCC827-ER, PC9-ER sh*NCAPG2*#2, and HCC827-ER sh*NCAPG2*#2 were detected by Hoechst staining using flow cytometry. PC9-ER sh*NCAPG2*#2 and HCC827-ER sh*NCAPG2*#2 had a significantly lower proportion of SP cells than PC9-ER and HCC827-ER (Figure 8E). This result demonstrated that the knockdown of *NCAPG2* resulted in the decreased stemness of the erlotinib resistance cells in LUAD, and *NCAPG2* plays a vital role in maintaining the stemness of LUAD.

Subsequently, 13 stemness marker genes (*THY1, SOX2, PROM1, POUSF1, KIT, ALCAM, ABCG2, ABCB1, ALDH1A1, CD44, ABCC1, EPCAM,* and *MYC*) were obtained from a review of the literature [12]. We performed a correlation analysis between *NCAPG2* and 13 lung cancer stemness markers using the TCGA database and found that *NCAPG2* was highly correlated with *ABCC1, MYC,* and *EPCAM* (Figure S4B). Therefore, we speculated that these three genes were downstream genes of *NCAPG2*. Then, the total RNA and the total protein of PC9-ER-control, HCC827-ER-control, PC9-ER-sh*NCAPG2*#2 and HCC827-ER-sh*NCAPG2*#2 were collected for RT-qPCR and Western blot to detect the expression of *ABCC1, MYC*, and *EPCAM*. RT-qPCR analysis showed lower expression levels of *ABCC1, MYC*, and *EPCAM* mRNA in the knockdown *NCAPG2* group compared to the control group (Figure S4C), suggesting that they may be downstream genes of *NCAPG2*. Additionally, Western blot showed that the knockdown of NCAPG2 decreased the protein expression of ABCC1, MYC, and EPCAM (Figure S4D). These results demonstrate that *NCAPG2* may affect the stemness of LUAD by affecting *ABCC1, MYC*, and *EPCAM*.

## 4. Discussion

Erlotinib is the first-line treatment drug for LUAD, but its drug resistance has been a major cause of poor prognosis in LUAD patients. Previous studies have reported that epidermal-mesenchymal transition (EMT) is responsible for EGFR-TKI resistance in LUAD and promotes lung cancer metastasis and tumor cell acquisition of stemness [31,32]. However, the relationship between erlotinib resistance and the stemness of LUAD requires further study. This study explores the relationship between erlotinib resistance and cancer stemness to find new therapeutic targets to guide the treatment of LUAD after erlotinib resistance.

Our study constructed an erlotinib resistance model through LASSO analysis of the GEO dataset, TCGA database, and CCLE database, which can accurately predict the patients’ responses to erlotinib. Based on this, we explored the potential molecular mechanisms of the model associated with erlotinib resistance. The GSVA showed that the high-resistance-score group could promote the stemness-related pathways, ‘MYC targets v1′ and ‘DNA repair’. Recent studies have found that CSCs have a strong ability to repair DNA damage in lung cancer [33]. Simon’s team also demonstrated that DNA damage repair systems make tumor cells resistant to chemotherapy in NCSLC [34]. Furthermore, Amy Schulze et al. confirmed that ‘MYC targets v1′ promotes breast cancer progression [35]. Subsequently, we also found that *NCAPG2* and *EIF2S3* were highly associated with stemness. Therefore, we determined whether erlotinib resistance was related to stemness in LUAD.

Immune cells play an essential role in drug resistance and stemness and the discovery of immune-related pathways by previous KEGG analysis using the high- and low-risk groups of the prognostic model. Therefore, we explored the correlation between immune cells and *NCAPG2* and *EIF2S3.* Immune infiltration analysis showed a negative correlation of *NCAPG2* and *EIF2S3* with most immune cells and a positive correlation with immune checkpoint molecules. Interestingly, *NCAPG2* and *EIF2S3* positively correlated with Th2 but negatively with Th1. Several researchers have investigated the role of Th2 in tumor promotion. According to Bretscher’s Th2-skewing hypothesis, large Th2 components in anti-tumor immune responses lead to tumor escape while Th1 mediates anticancer immunity in mice [36,37]. Similarly, Astri Frafjord’s team showed that Th2 is dominant in the immune landscape of human primary lung tumors [38]. Tomohide Tatsumi’s team found elevated Th2 in renal cell carcinoma [39]; Elena Tassi et al. confirmed a tumor-related Th2-immune deviation in pancreatic cancer [40]. Moreover, Th2 cells were thought to promote tumor progression by secreting cytokines such as IL-4 and IL-13 [41]. Based on previous research, we conjecture that tumor immune escape mediated by *NCAPG2* and *EIF2S3* may be involved in LUAD erlotinib resistance and stemness. A bioinformatics study found that *NCAPG2* was more correlated with the mRNAsi than *EIF2S3*. We selected *NCAPG2* for in vitro study based on this finding. This means that in future we will focus on *NCAPG2* and explore the underlying molecular mechanism.

*NCAPG2* is a component of the condensin II complex and plays a vital role in cell mitosis [42]. Many studies have shown that *NCAPG2* is highly expressed in tumors and involved in tumor proliferation, metastasis, and invasion. *NCAPG2* facilitates glioblastoma cell malignancy and xenograft tumor growth via HBO1 activation by phosphorylation [43]. *NCAPG2* overexpression drives Hepatocellular carcinoma (HCC) proliferation and metastasis by activating the STAT3 and NF-κB/miR-188-3p pathways [44]. *NCAPG2* promotes LUAD cell proliferation by regulating the G2/M phase [22]. Previous studies have also shown that *NCAPG2* is mainly involved in signaling pathways such as G2M checkpoint, DNA repair, and oxidative phosphorylation [45]. Coincidentally, this is consistent with the GSVA results of our drug resistance model. According to these results, we speculate that *NCAPG2* might influence the development of erlotinib resistance and stemness in LUAD. Our results from the GSVA analysis and functional experiments with *NCAPG2* also validated this hypothesis. In our study, we found that the high expression of *NCAPG2* activates the mTORC1 signaling pathway. Previously, Ye et al. demonstrated that *TM4SF1* induced chemoresistance in NSCLC by regulating the DDR1/ERK/Akt-mTOR pathway [46]. Chen et al. found that the PI3K/Akt/mTOR signaling pathway promoted the proliferation and survival of colon cancer stem cells [47]. Excessive activation of mTORC1 has also been shown to promote tumor formation, proliferation, and metastasis [48]. Therefore, we speculated that *NCAPG2* may function through the mTOCR1 signaling pathway. Interestingly, we also found that increased *NCAPG2* expression enhanced MYC-related pathways. As one of the transcription factors driving the pluripotency of embryonic stem cells, MYC is also considered to be one of the stemness marker genes of LUAD [24]. This suggested that NCAPG2 might affect the stemness of LUAD by affecting MYC-related pathways. Based on these findings, we hypothesized that *NCAPG2* might affect stemness and erlotinib resistance in LUAD through the mTOR and MYC target pathways. We found that the knockdown of *NCAPG2* decreased the expression of *ABCC1, MYC*, and *EPCAM* through the experiments. So, we speculate that *NCAPG2* may maintain the stemness of LUAD and promote erlotinib resistance through *ABCC1, MYC*, and *EPCAM*. However, the specific mechanism needs to be further explored.

Although our study suggests that *NCAPG2* plays an important role in maintaining stemness and promoting erlotinib resistance in LUAD, there are still some limitations. Our analysis is based on public databases without our own clinical samples; so, there might be some deviations. Moreover, the clinical medication guide for erlotinib states that the clinical dose is 150 mg/day, which is greater than the concentration of erlotinib-resistant strains we constructed. Therefore, further studies are needed regarding the underlying mechanism between erlotinib resistance and stemness in LUAD.

## 5. Conclusions

An erlotinib resistance model was constructed based on erlotinib resistance genes, which could predict the patients’ responses to erlotinib. In addition, *NCAPG2* was found to maintain the stemness and promote erlotinib resistance in lung adenocarcinoma. This study might provide new insights into diagnosing and treating LUAD with erlotinib resistance.

## Figures and Tables

**Table 1:** Univariate and multivariate Cox regression.

Characteristics	Total (N)	Univariate Analysis		Multivariate Analysis	
		Hazard Ratio (95% CI)	*p* Value	Hazard Ratio (95% CI)	*p* Value
*NCAPG2*	496	1.266 (1.088–1.474)	0.002	1.203 (1.028–1.409)	0.022
*EIF2S3*	496	1.380 (1.092–1.744)	0.007	1.547 (1.185–2.020)	0.001
pathologic_stage	496				
Stage I	268				
Stage II	120	2.383 (1.657–3.428)	<0.001	1.868 (1.126–3.100)	0.016
Stage III	83	3.134 (2.145–4.579)	<0.001	2.550 (1.450–4.486)	0.001
Stage IV	25	3.761 (2.168–6.524)	<0.001	3.332 (1.815–6.114)	<0.001
pathologic_N	496				
N0	319				
N1	177	2.477 (1.845–3.325)	<0.001	1.304 (0.823–2.066)	0.258
pathologic_M	496				
M0	330				
M1	166	1.044(0.758–1.439)	0.790		

## Data Availability

The following information was supplied regarding data availability: The data is available at the TCGA database (https://portal.gdc.cancer.gov/ (accessed on 23 March 2022)), CCLE (https://portals.broadinstitute.org/ccle/data (accessed on 23 March 2022)), GDSC (https://www.cancerrxgene.org/ (accessed on 23 March 2022)), and NCBI GEO: GSE31625, GSE35603, GSE72094, GSE10072, and GSE30219.

## Supplementary Materials

The following supporting information can be downloaded at: https://www.mdpi.com/article/10.3390/cancers14184395/s1, Figure S1: workflow diagram, Figure S2: identification of biological pathways associated with the risk of prognostic signature, Figure S3: single-cell RNA sequencing (scRNA-seq) analysis using GSE134839, Figure S4: relationship between NCAPG2 and stemness markers in LUAD, Table S1: Primers for qPCR.

## Abbreviations

EGFR-TKI	Growth factor receptor tyrosine kinase inhibitors
CSCs	Cancer stem cells
LUAD	Lung adenocarcinoma
NSCLC	Non-small cell lung cancer
SCLC	Small cell lung cancer
OS	Overall survival
DEGs	Differentially expressed genes
KM	Kaplan–Meier
GO	Gene Ontology
KEGG	Kyoto Encyclopedia of Genes and Genomes
IC50	50% inhibitory concentration
GSVA	Gene Set Variation Analysis
mRNAsi	The stemness index based on mRNA expression
scRNA-seq	Single-cell RNA sequencing
