# Supplementary material for: NCAPG2 Maintains Cancer Stemness and Promotes Erlotinib Resistance in Lung Adenocarcinoma
Source: Cancers (Basel). 2022 Sep 9;14(18):4395. doi: 10.3390/cancers14184395 (PMC9497119; doi:10.3390/cancers14184395)

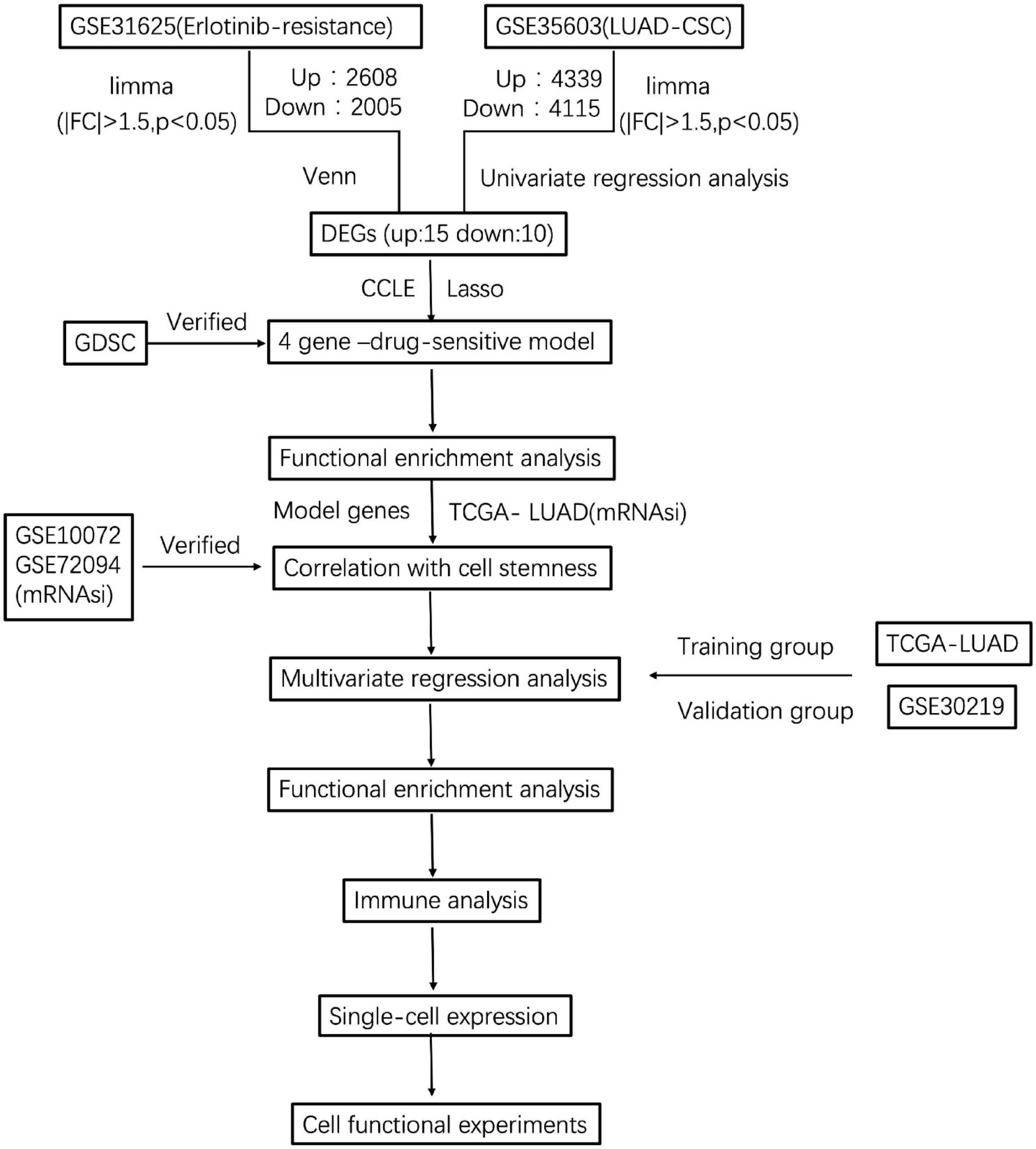

Figure S1. Workflow Diagram.

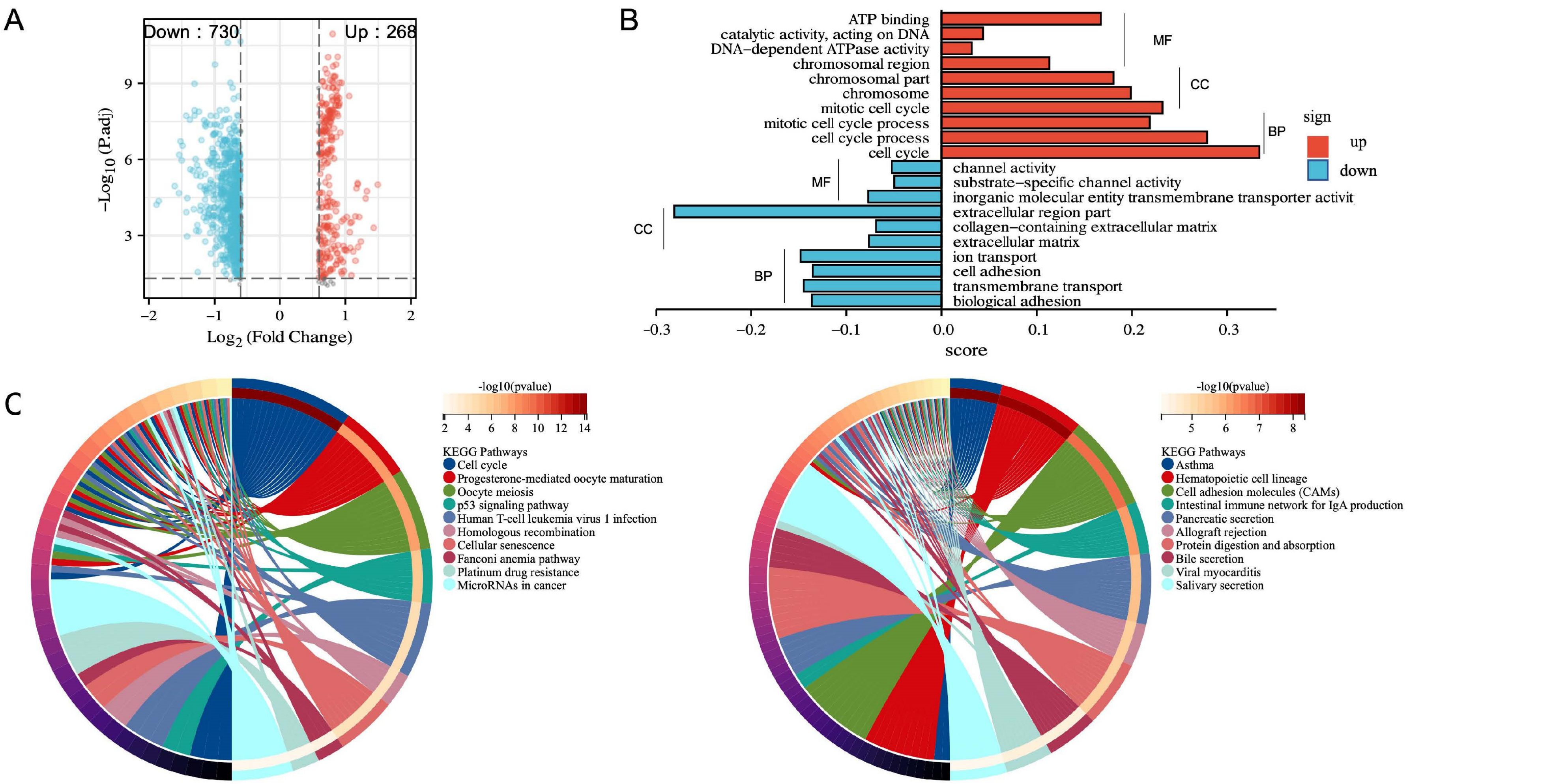

Figure S2. Identification of biological pathways associated with the risk of prognostic signature. (**A**) DEGs between different risk groups. (**B**) GO analysis of DEGs between different risk groups into three functional groups, including BP, CC and MF. (**C**) KEGG between the high-risk and low-risk groups of TCGA cohort. BP: biological process.

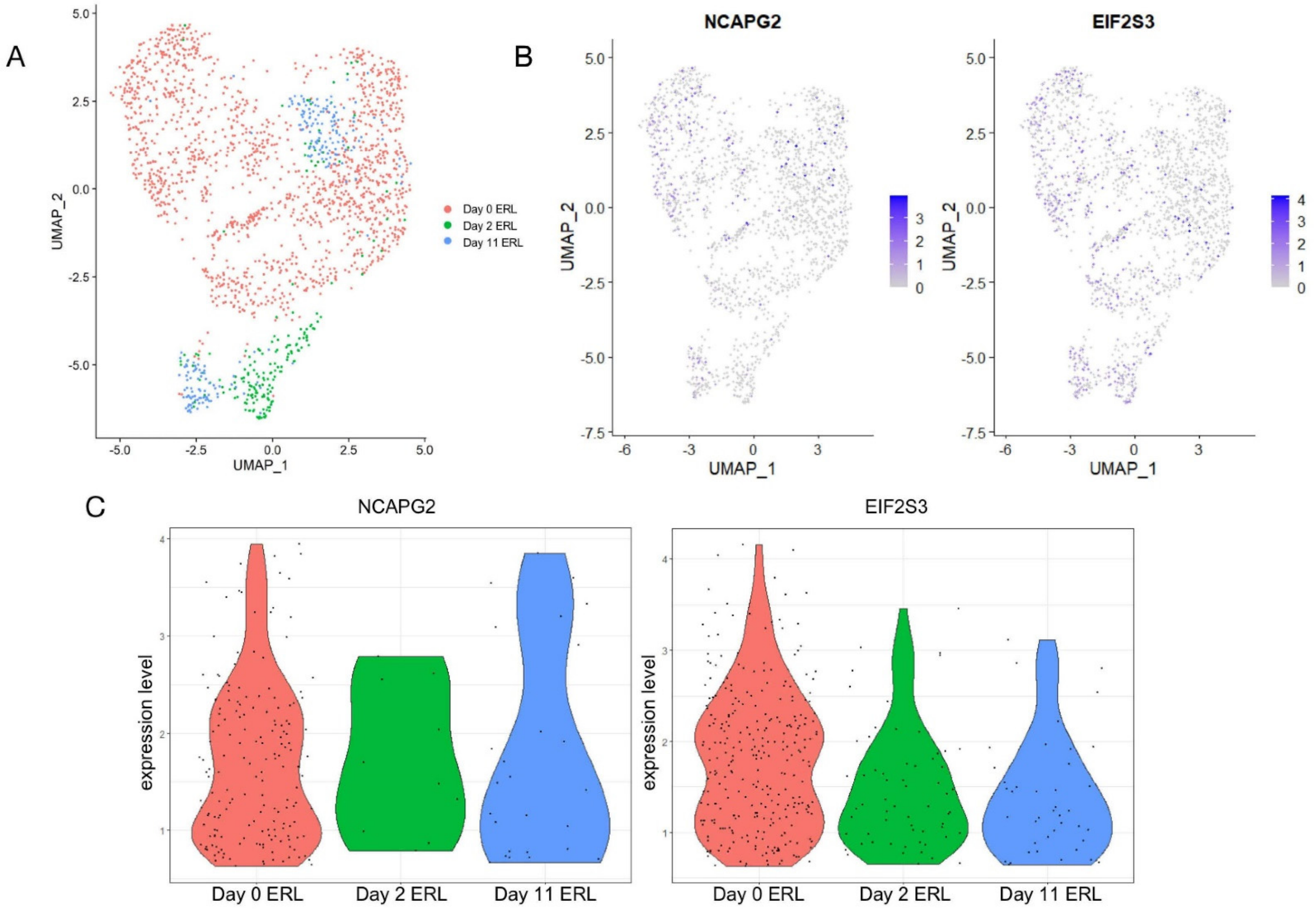

Figure S3. Single-cell RNA sequencing (scRNA-seq) analysis using GSE134839. (**A**) Visualization of scRNA-seq in three datasets using UMAP. (**B**,**C**) UMAP plots and violin plots showing the expression of NCAPG2 and EIF2S3 in cell populations.

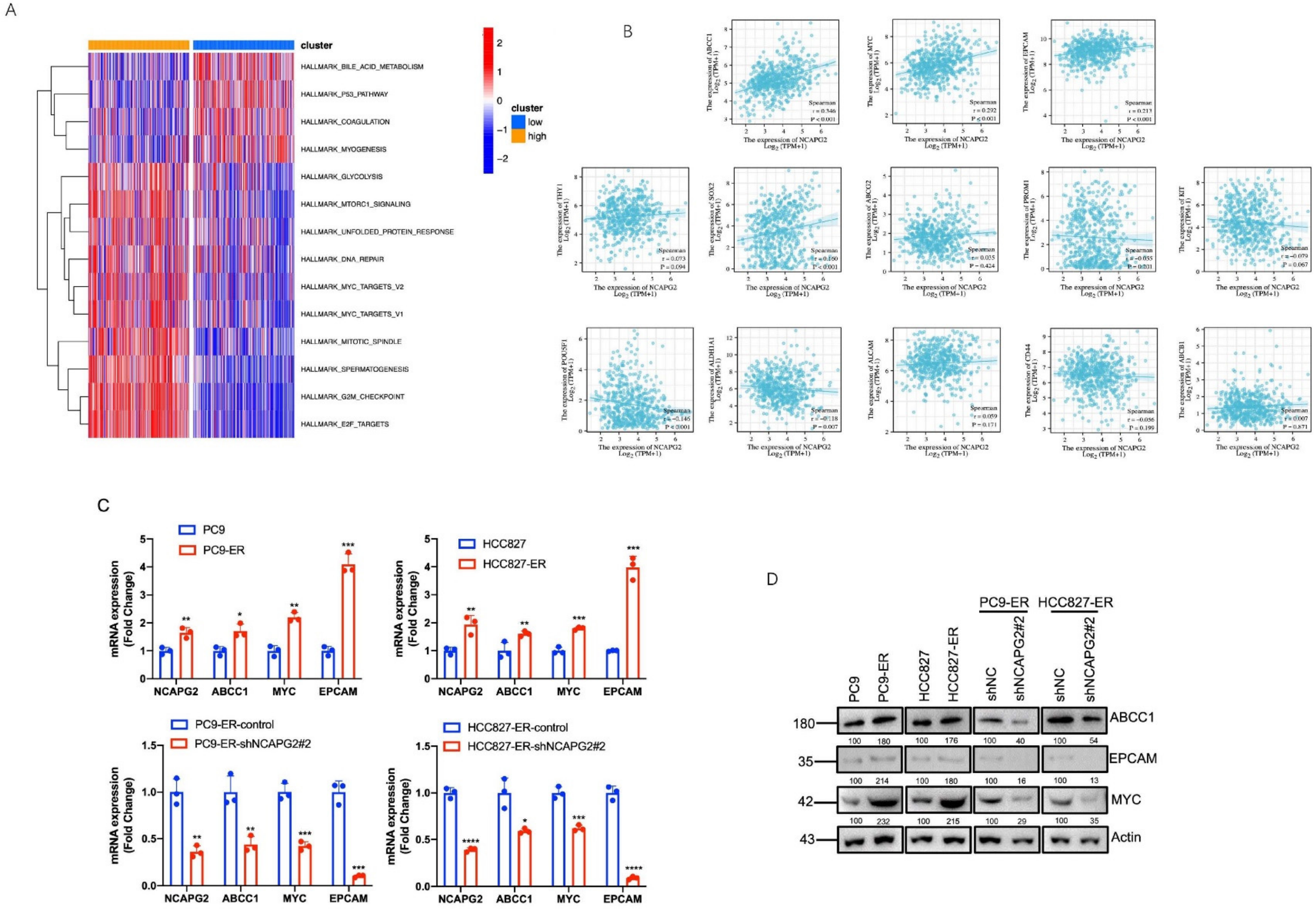

Figure S4. Relationship between NCAPG2 and stemness markers in LUAD. (**A**) GSVA of NCAPG2 expression. (**B**) Scatter plot shows the correlation between NCAPG2 and 13 stemness markers in lung cancer in TCGA database. (**C**) qPCR displays the expression of 3 stemness markers (ABCC1, EPCAM, MYC) in PC9-ER, HCC827-ER, PC9-ER control, PC9-ER shNCAPG2#2, HCC827-ER control and HCC827-ER shNCAPG2#2. (**D**) Western blot displays the expression of 3 stemness markers (ABCC1, EPCAM, MYC) in PC9-ER, HCC827-ER, PC9-ER control, PC9-ER shNCAPG2#2, HCC827-ER control and HCC827-ER shNCAPG2#2.

Table S1. Primers for qPCR.

Primer	Forward Sequence	Reverse Sequence
NCAPG2	5′-GGAACTGGCATTTGACACGAGC-3′	5′-GCTGCTCTAACAATGGGTGGCT-3′
ABCC1	5′-CCGTGTACTCCAACGCTGACAT-3′	5′-ATGCTGTGCGTGACCAAGATCC-3′
MYC	5′-CCTGGTGCTCCATGAGGAGAC-3′	5′-CAGACTCTGACCTTTTGCCAGG-3′
EPCAM	5′-GCCAGTGTACTTCAGTTGGTGC-3′	5′-CCCTTCAGGTTTTGCTCTTCTCC-3′
β-actin	5′-CACCATTGGCAATGAGCGGTTC-3′	5′-AGGTCTTTGCGGATGTCCACGT -3′

uncropped blots

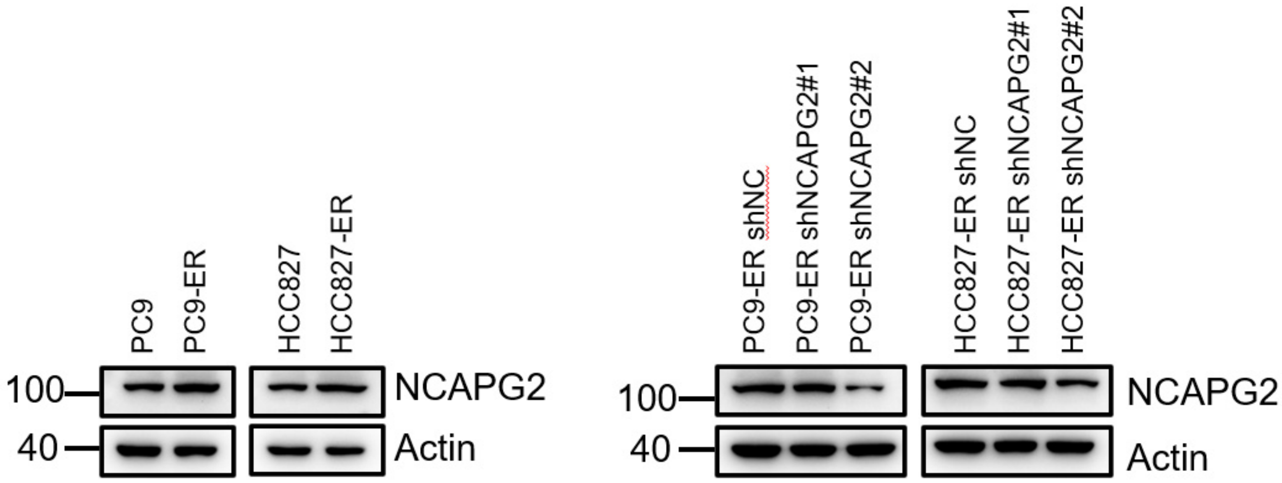

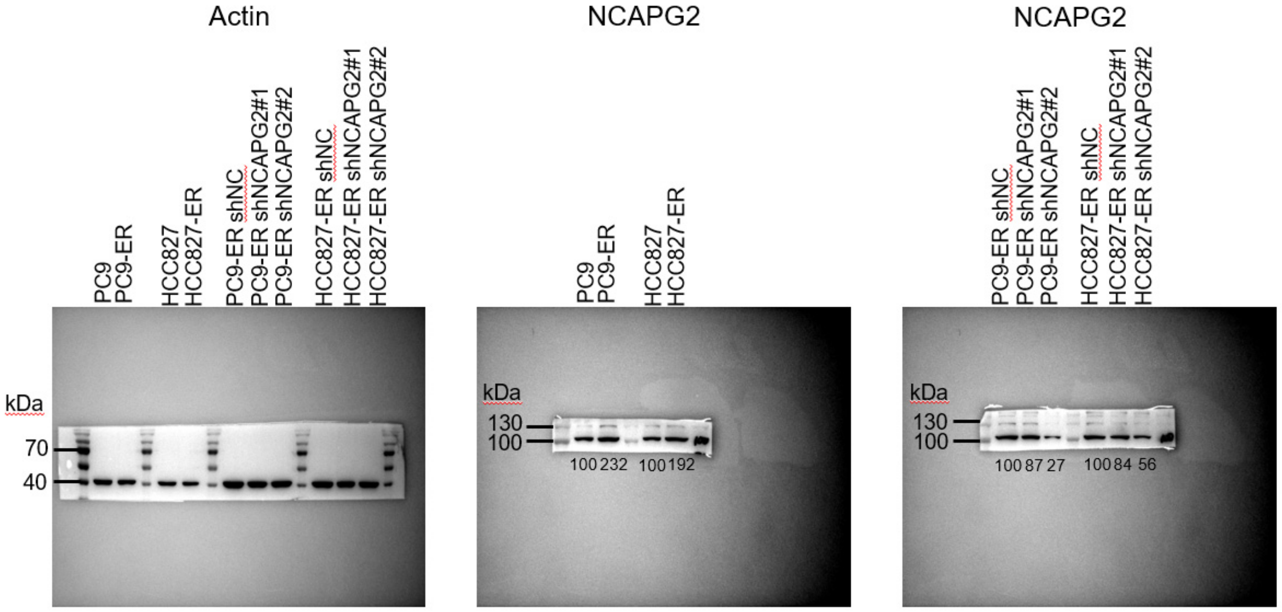

Supplement: Supplementary file 1 [file cancers-14-04395-s001.zip › cancers-1839290-supplementary.pdf]
